# The effect of antipsychotic medication on sexual function and serum prolactin levels in community-treated schizophrenic patients: results from the Schizophrenia Trial of Aripiprazole (STAR) study (NCT00237913)

**DOI:** 10.1186/1471-244X-8-95

**Published:** 2008-12-22

**Authors:** Linda Hanssens, Gilbert L'Italien, Jean-Yves Loze, Ronald N Marcus, Miranda Pans, Wendy Kerselaers

**Affiliations:** 1 Bristol-Myers Squibb Company, Parc de l'Alliance, Avenue de Finlande, 8, B-1420 Braine-l'Alleud, Belgium; 2 Bristol-Myers Squibb Company, 5 Research Parkway, Wallingford, CT 06492, USA; 3 Otsuka Pharmaceutical France SAS, "Les Colonnades" -4, rue Henri Sainte Claire Deville, 92500 Rueil-Malmaison, France

## Abstract

**Background:**

The aim of this paper is to evaluate the effect of antipsychotics for the treatment of schizophrenia in a community based study on sexual function and prolactin levels comparing the use of aripiprazole and standard of care (SOC), which was a limited choice of three widely used and available antipsychotics (olanzapine, quetiapine or risperidone) (The Schizophrenia Trial of Aripiprazole [STAR] study [NCT00237913]).

**Method:**

This open-label, 26-week, multi-centre, randomised study compared aripiprazole to SOC (olanzapine, quetiapine or risperidone) in patients with schizophrenia (DSM-IV-TR criteria). The primary effectiveness variable was the mean total score of the Investigator Assessment Questionnaire (IAQ) at Week 26. The outcome research variables included the Arizona Sexual Experience scale (ASEX). This along with the data collected on serum prolactin levels at week 4, 8, 12, 18 and 26 will be the focus of this paper.

**Results:**

A total of 555 patients were randomised to receive aripiprazole (n = 284) or SOC (n = 271). Both treatment groups experienced improvements in sexual function from baseline ASEX assessments. However at 8 weeks the aripiprazole treatment group reported significantly greater improvement compared with the SOC group (p = 0.007; OC). Although baseline mean serum prolactin levels were similar in the two treatment groups (43.4 mg/dL in the aripiprazole group and 42.3 mg/dL in the SOC group, p = NS) at Week 26 OC, mean decreases in serum prolactin were 34.2 mg/dL in the aripiprazole group, compared with 13.3 mg/dL in the SOC group (p < 0.001).

**Conclusion:**

The study findings suggest that aripiprazole has the potential to reduce sexual dysfunction, which in turn might improve patient compliance.

## Background

All antipsychotic medications have the potential to cause hyperprolactinaemia, as the inhibition of dopamine release effectively removes the negative feedback loop for prolactin secretion from the anterior pituitary gland [1]. Elevated serum prolactin levels have been shown to have profound effects on reproductive health and sexual function, including hypogonadism, decreased libido in both sexes, amenorrhoea and infertility in women and low sperm count and reduced muscle mass in men [2]. In addition, there is evidence to suggest a link between hyperprolactinaemia and reduced bone mineral density – a study of women with hyperprolactinaemia showed a 15–30% reduction in bone mineral density compared with healthy women [3]. There is also a suspected link between elevated plasma prolactin levels and breast cancer, although the causal relationship in this link remains to be established [1].

Although psychotic illness can be associated with sexual problems there have been considerable reports in the literature of sexual dysfunction related to antipsychotic use being a significant issue of patients, which may have a negative impact on the patient's quality of life and adherence to treatment [4,5]. Clinical studies have demonstrated that with the exception of risperidone, atypical antipsychotics have significantly less impact on serum prolactin compared with conventional antipsychotics [6-10], and are also less likely to cause sexual dysfunction [11-13]. In addition Gopalakrishnan et al [5] has shown that it is possible to successfully treat antipsychotic sexual dysfunction with sildenafil including patients exhibiting raised prolactin levels. In this study 74% (23 patients) of the sildenafil treatment group felt that in future they would use this treatment compared to 26% of the placebo group. Whilst short-term clinical studies with aripiprazole have shown comparable serum prolactin levels to placebo [14], the effects of aripiprazole on serum prolactin and sexual function over a longer period of time have yet to be investigated.

The Schizophrenia Trial of Aripiprazole (STAR) study is a naturalistic effectiveness study conducted with the aim of providing critical information on the relationships between the condition, the medication, and the patients in their everyday care setting. Following an initial review of atypical antipsychotics in 2002, the UK National Institute of Health and Clinical Excellence (NICE) found a lack of 'real-world' data such as patient-reported outcomes, patient preferences, and treatment regimens based on the clinician's judgment [15]. For subsequent reviews, NICE has acknowledged that whilst randomised clinical trials are of restricted applicability to the general patient population due to their limited duration and number of variables measured, naturalistic studies such as STAR allow evaluations of treatment effectiveness using less selective patient criteria and a wider spectrum of effectiveness variables (such as safety, tolerability, symptom relief or worsening, patient preference for medication, quality of life, and cost-effectiveness) [16,17].

The primary objective of the STAR study was to evaluate the effectiveness of a 26-week treatment of aripiprazole versus standard of care (SOC), which was a limited choice of three widely used and available antipsychotics (olanzapine, quetiapine or risperidone), in patients treated for schizophrenia in a community health or hospital-based outpatient setting. Outcomes research variables included quality of life and sexual function as secondary outcomes, and the safety evaluation included monitoring of serum prolactin levels. This paper will discuss specifically the findings with respect to sexual function in light of the serum prolactin analysis.

## Methods

### Patients

Patients at 98 centres in 12 countries throughout Europe were enrolled in the study between July 2004 and June 2005. The study protocol complied with the Declaration of Helsinki and Good Clinical Practices (GCP) guidelines and was approved by the Institutional Review Board (IRB)/Independent Ethics Committee (IEC) at the individual study centres. Prior to study enrolment, informed written consent was obtained from all patients.

Eligible for inclusion in the study were male and female patients aged 18 to 65 years with a diagnosis of schizophrenia (DSM-IV-TR criteria) treated within a community mental health or hospital-based outpatient centre, for whom a change of antipsychotic medication was indicated due to lack of tolerability and/or symptom control as judged by the clinician.

Key exclusion criteria included acute psychotic symptoms requiring hospitalisation, risk of committing suicide, a diagnosis of schizoaffective disorder, bipolar disorder, depression with psychotic symptoms, or organic brain syndrome. Patients were also excluded if they were considered treatment resistant, had had a significant psychoactive substance use disorder within 3 months prior to screening, or had any history of neuroleptic malignant syndrome, epilepsy, seizures, abnormal electroencephalogram, severe head injury, or stroke.

### Study design

The design and methodology of the STAR study have been presented in detail elsewhere; hence only a summary will be provided here [18].

The STAR study was designed as a multi-centre, randomised, naturalistic, open-label study to compare aripiprazole treatment with SOC treatment (limited to a choice of olanzapine, quetiapine or risperidone). Eligible patients were randomised to receive treatment with aripiprazole or SOC for a period of 26 weeks. Patients randomised to the SOC group received one of three selected atypical antipsychotics: olanzapine, quetiapine, or risperidone, based on the investigator's/clinician's judgment as to the optimal treatment for the individual patient and taking into account the patients' previous response to antipsychotic medication.

A cross-titration period of up to 14 days was permitted following the randomisation, with complete discontinuation of any pre-study antipsychotic medication by Day 15. During the 26-week open-label treatment phase, study visits occurred at Weeks 2, 4, 8, 12, 18 and 26 to assess the effectiveness of study treatment.

Patients randomised to the aripiprazole group received a starting dose of 15 mg/day followed by a daily dose of 10–30 mg/day, as judged by the investigator, administered orally once daily independent of meals. Patients randomised to the SOC group received either olanzapine, starting at 10 mg once daily followed by a daily dose of 5–20 mg/day; quetiapine, starting with 50 mg/day on Day 1, 100 mg/day on Day 2, 200 mg/day on Day 3, and 300 mg/day on Day 4, then followed by an individually titrated dose of 800 mg/day maximum; risperidone, starting with 2 mg/day on Day 1, 4 mg/day on Day 2, then a daily dose of 2–16 mg/day; or dosages according to the approved local labelling for each of the three medications.

Benzodiazepines and anticholinergics were allowed during the study if deemed necessary by the investigator. Other psychotropic medications such as antidepressants and mood stabilisers were allowed if the patient was already receiving these medications when entering the study. No additional antipsychotics were allowed during the study.

### Evaluations

#### Effectiveness assessments

Full details of the Investigator Assessment Questionnaire (IAQ) and Clinical Global Impression (CGI) Improvement (CGI-I) and Severity (CGI-S) assessments have been published elsewhere [18]. Briefly, the clinical effectiveness of the study treatments was assessed through the IAQ. The primary effectiveness variable in the trial was the mean total score of the 10-item IAQ at Week 26, last observation carried forward (LOCF).

Secondary effectiveness variables included the CGI-I and CGI-S scales. IAQ, CGI-I and CGI-S scores were obtained at Weeks 2, 4, 8, 12, 18, and at the end of Week 26.

#### Outcomes research assessments

The STAR study outcomes research evaluation comprised the following tools: the Preference of Medicine (POM) questionnaire, the Quality of Life Scale (QLS), the Client Socio-demographic and Service Receipt Inventory – European version (CSSRI-EU), EuroQoL-5D (EQ-5D), Impact of Weight on Quality of Life (IWQoL-Lite), and the Arizona Sexual Experience Scale (ASEX). This paper will report and discuss the findings of the ASEX analysis; the other analyses will be reported separately.

##### Arizona Sexual Experience Scale (ASEX)

The ASEX is a 5-item scale to assess sexual dysfunction amongst psychiatric patients [19]. The ASEX rates sex drive, arousal, vaginal lubrication/penile erection, ability to reach orgasm, and satisfaction from orgasm. Male and female patients are assessed separately. Possible Total Score ranges from 5 to 30, with the higher score indicating more patient sexual dysfunction. The scale could be either self-administered or clinician-administered at the discretion of the treating clinician. The scale is applicable to patients regardless of the availability of a sexual partner. The ASEX was administered at baseline and at Weeks 8, 18, with a final assessment at the end of Week 26 or at the time of premature discontinuation.

#### Safety assessments

The safety variables of interest for this paper are the change from baseline in serum prolactin levels throughout the study, and the proportion of patients with potentially clinically significant elevations in prolactin levels. Prolactin serum levels were assessed by the Abbott AxSYM system prolactin assay – a Microparticle Enzyme Immunoassay (MEIA) for the quantitative measurement of serum prolactin. The normal range for this test was defined as 1.6–18.8 mg/dL for men and 1.4–24.2 mg/dL for women. The Statistical Analysis Plan for the STAR study defines a clinically significant lab value for prolactin as any value above the upper limit of normal. In addition to serum prolactin, the safety analysis comprised the incidence of adverse events (AEs), including treatment-emergent AEs, AEs leading to discontinuation, treatment-emergent serious AEs, and treatment-emergent EPS-related AEs. Additional safety endpoints included the proportion of subjects with potentially clinically relevant vital signs/laboratory findings as defined by the Food and Drug Administration Division of Neuropharmacological Drug Products [20]

### Statistical procedures

#### Sample size and power

It was estimated that 556 patients would have to be randomised to obtain 500 evaluable patients (250 on aripiprazole and 250 on SOC), assuming that 90% of the randomised patients would be evaluable in the primary endpoint. This sample size would yield 95% statistical power with a true difference in the primary endpoint of 2. This assumed a standard deviation of 6.2 and a 2-sided t-test at the 0.05 level of significance. This sample size would also allow for meaningful analysis of secondary endpoints for which it is difficult to obtain reliable estimates of variance, and for exploratory analysis to estimate the effect size of aripiprazole relative to the individual SOC items.

#### Data sets

The safety sample comprised all randomised patients who took at least one dose of study medication. The effectiveness sample included all patients in the safety sample who had at least one post-baseline effectiveness evaluation (IAQ or CGI). The outcome research sample, which included assessments of quality of life and medication preference, was comprised of all patients in the safety sample who had at least one post-baseline outcome research evaluation (including ASEX). LOCF was the primary analysis used for the effectiveness research sample (IAQ, CGI-I, CGI-S), whereas observed cases (OC) was the primary analysis used for the outcomes research sample (ASEX).

#### Analyses

The results from the ASEX evaluation were subject to analysis of covariance (ANCOVA) with respect to the mean change from baseline in ASEX Total Score. The ANCOVA model included treatment group (aripiprazole, SOC) and gender as main effects, with the baseline score as a covariate. Each additional OC week and each LOCF week was analysed similarly. For the analysis of LOCF data, country was also included as a main effect within the model. In addition, the baseline ASEX Total Score was analysed with an ANCOVA model including treatment group and gender (and country for the LOCF data set).

The proportions of patients with potentially clinically relevant laboratory test values during the 26-week period were summarised by treatment group. The proportions of patients with potentially clinically relevant metabolic parameters during the 26-week period were analysed using a Cochran-Mantel-Haenszel test controlling for the presence or absence of baseline abnormality.

The change from baseline in serum prolactin was analysed using an ANCOVA model which included treatment group as main effect and baseline value as the covariate. Baseline serum prolactin levels were analysed with an ANCOVA model which included treatment group.

Correlation between changes in prolactin and in ASEX Total score were calculated in a post-hoc analysis using Spearman rank-order partial correlation controlling for antipsychotic taken and gender. The antipsychotic taken was preferred in this analysis over the treatment group as the effect on prolactin may be different for each antipsychotic. In another post-hoc analysis, the change from baseline in ASEX Total score (LOCF) was analysed using the ANCOVA model as described above, but with on treatment CGI-Improvement added as covariates to assess whether the ASEX advantage is specific and not the result of the patients overall improvement. Although such an analysis may suggest a residual treatment effect on the ASEX Total Score that can not be attributed to the patient's global improvement, any analysis that adjusts for post-randomization covariates must be considered speculative in nature.

## Results

The demographics, primary efficacy results and safety findings of the STAR study have been presented in detail elsewhere [18]. This paper will present the results of the patient-reported secondary endpoint ASEX and the safety endpoint of serum prolactin levels.

### Patient disposition and demographics

A total of 593 patients were enrolled into the study and 555 patients were randomised to receive study treatment with aripiprazole (n = 284) or SOC (n = 271) for 26 weeks. Of patients randomised to receive SOC, 75 patients received olanzapine, 110 patients received quetiapine, and 81 patients received risperidone. Five patients in the SOC group were randomised, but never received study medication. Table 1 shows the baseline demographic characteristics of the randomised patient population including their previous medication. Patient disposition data are summarised in Table 2.

**Table 1:** Baseline demographic characteristics of the randomised patient population [18].

**Variable**	**Aripiprazole (N = 284)**	**All SOC (N = 271)**	**Total (N = 555)**
**Age, years**			
Mean	38.1	38.8	38.5
Median	36.5	37.0	37.0
			
SE	10.8	11.1	10.9
			
**Gender, n (%)**			
Male	169 (59.5)	163 (60.1)	332 (59.8)
Female	115 (40.5)	108 (39.9)	223 (40.2)
**Race, n (%)**			
White	274 (96.5)	262 (96.7)	536 (96.6)
Black	3 (1.1)	1 (0.4)	4 (0.7)
Asian	3 (1.1)	6 (2.2)	9 (1.6)
Other	4 (1.4)	2 (0.7)	6 (1.1)
Weight, kg			
Mean	80.4	81.0	80.7
Median	79.0	79.0	79.0
SE	17.5	17.0	17.2
Missing		2	2
**Schizophrenia type, n (%)**			
Disorganised	24 (8.5)	32 (11.8)	56 (10.1)
Catatonic	3 (1.1)	4 (1.5)	7 (13)
Paranoid	189 (66.5)	172 (63.5)	361 (65.0)
Residual	31 (10.9)	36 (13.3)	67 (12.1)
Undifferentiated	37 (13.0)	27 (10.0)	64 (11.5)
**Age at time of first hospitalisation, years**			
Mean	28.3	28.7	28.5
Median	27.0	27.0	27.0
SE	8.5	8.7	8.6
Missing	35	26	61
**Previous medication, N (%)**			
Any Antipsychotic Medication*	273 (96.1)	267 (98.5)	540 (97.3)
Olanzapine	75 (26.4)	85 (31.4)	160 (28.8)
Quetiapine	15 (5.3)	17 (6.3)	32 (5.8)
Risperidone	66 (23.2)	69 (25.5)	135 (24.3)

* The remaining patients not accounted for by olanzapine, quetiapine and risperidone received a range of antipsychotic medication including amisulpride, benperidol, bromperidol, chlorpromazine, chlorprothixene, clozapine, cyamemazine, flupenthixol, fluphenazine, haloperidol, loxapine, melperone, methotrimeprazine, perazine, periciazine, perphenazine, pipamperone, pipothiazine, sertindole, sulpiride, thioridazine, trifluoperazine, ziprasidone, zotepine, zuclopenthixol

**Table 2:** Summary of patient disposition [18].

**Patient Status**	**Number of patients (%)**		
	**Aripiprazole**	**All SOC**	**Total**
Enrolled			593
Randomised	284	271	555
Discontinuation from treatment phase	120 (42.3)	105 (38.7)	225 (40.5)
Lack of efficacy	33 (11.6)	20 (7.4)	53 (9.5)
Adverse event	57 (20.1)	43 (15.9)	100 (18.0)
Withdrew consent	8 (2.8)	18 (6.6)	26 (4.7)
Death	1 (0.4)	2 (0.7)	3 (0.5)
Lost to follow-up	6 (2.1)	6 (2.2)	12 (2.2)
Poor or non-compliance	10 (3.5)	10 (3.7)	20 (3.6)
Pregnancy	2 (0.7)	2 (0.7)	4 (0.7)
Non longer meets study criteria	2 (0.7)	3 (1.1)	5 (0.9)
Other	1 (0.4)	1 (0.4)	2 (0.4)
Completed treatment phase	164 (57.7)	166 (61.3)	330 (59.5)
Randomised, but did not receive study medication	2	5	7

The most common primary reason for a change in medication in accordance with the study inclusion criteria were poorly controlled positive (aripiprazole 32%, SOC 23%) and negative symptoms (aripiprazole 25%, SOC 32%), followed by weight gain (aripiprazole 12%, SOC 12%). The mean daily dose of study medication at the end of the treatment period was 18.7 mg aripiprazole, 12.5 mg olanzapine, 386.8 mg quetiapine, and 4.6 mg risperidone.

### Clinical effectiveness and outcomes

#### Primary efficacy outcomes

The results of the primary effectiveness analysis (IAQ, GCI-I and CGI-S scores) in the STAR study have been described in detail elsewhere [18]. However in summary the aripiprazole treatment group (n = 268) resulted in significantly better effectiveness than SOC (n = 254; P < 0.001; week 26 LOCF) as evidenced by the IAQ total score beginning at the first assessment point (week 4) and this was sustained through to week 26.

#### Patient-reported secondary outcome variables: sexual function (ASEX)

**Figure 1:**
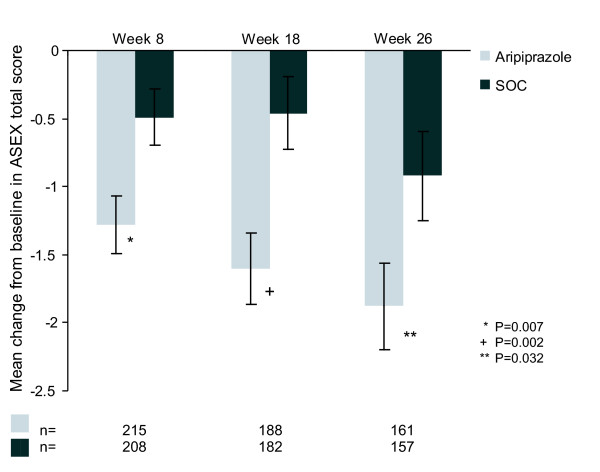
**Mean change from baseline in ASEX Total score (OC)**.

Both treatment groups experienced improvements in sexual function from baseline as assessed by ASEX; the improvement was significantly greater in the aripiprazole group compared with the SOC group (Figure 1). The difference in favour of aripiprazole was statistically significant from Week 8 (first assessment point) onwards. At 26 weeks, the ASEX Total Score had dropped by 1.88 points from 19.27 at baseline, compared with a drop of 0.92 from 19.7 at baseline in the SOC group (p = 0.032; OC).

**Figure 2:**
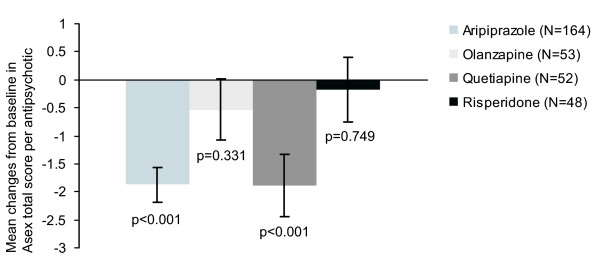
**Mean change from baseline in ASEX Total score at week 26 by drug (OC)**.

Further analyses were performed on each antipsychotic taken. However, the results of this sub-analysis should be treated with care as the investigator could determine the antipsychotic taken in the SOC group. Figure 2 shows the results for each agent at week 26 (OC data) demonstrating that of the SOC group quetiapine had a greater improvement on ASEX Total score than olanzapine or risperidone. At baseline all groups had similar ASEX Total scores.

There was no interaction found between treatment and gender in the analysis of the ASEX Total Score. However when pooling the SOC and aripiprazole group, male patients exhibited an improvement in ASEX Total score of -1.53 (SE 0.31) from a baseline score of 17.93 compared to an improvement of -0.71 (SE 0.41) in the female patients (baseline score 21.90), suggesting that for the male patients sexual dysfunction was more affected by a change of medication.

In a post-hoc analysis, the change from baseline in ASEX Total Score was also adjusted for CGI-I, and a statistically significant treatment difference was still observed (-1.47 for aripiprazole versus -0.67; p = 0.024).

### Safety

#### Adverse events

The results of the safety analysis have been reported in detail elsewhere [18]. In summary, the incidence of treatment-emergent AEs was similar in both treatment groups (77% in the aripiprazole group versus 71% in the SOC group). The most common AEs (≥ 10%) were insomnia, anxiety, headache, and nausea in the aripiprazole group, and anxiety and somnolence in the SOC group.

#### Serum prolactin levels

**Figure 3:**
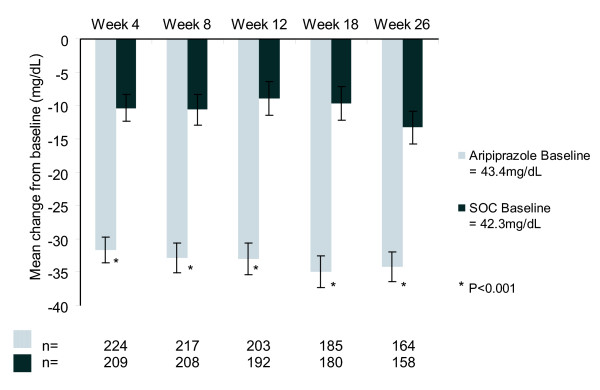
**Mean change from baseline in serum prolactin concentration (OC)**.

Baseline mean serum prolactin levels were similar in the two treatment groups (43.4 mg/dL in the aripiprazole group and 42.3 mg/dL in the SOC group, p = NS). At week 26, mean decreases in serum prolactin were 34.2 mg/dL in the aripiprazole group, compared with 13.3 mg/dL in the SOC group (p < 0.001; OC) (Figure 3). The maximum on-treatment effect on serum prolactin levels in the safety sample was a mean reduction of 28.8 mg/dL in the aripiprazole, versus a mean increase of 0.3 mg/dL in the SOC group (p < 0.001).

Potentially clinically relevant elevations in prolactin levels during the 26-week period were seen in a significantly higher proportion of patients in the SOC group (136 patients [54.4%]) compared with the aripiprazole group (45 patients [16.8%]).

**Figure 4:**
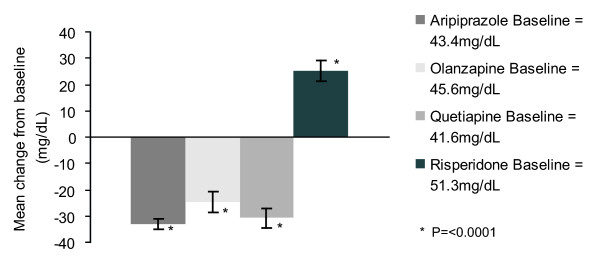
**Mean change from baseline in serum prolactin concentration at week 26 by drug (OC)**.

When interpreting the results of these sub analyses, it should be considered that the investigators could choose the antipsychotic treatment in the SOC group. As can be seen in figure 4, the patients treated with aripiprazole, olanzapine and quetiapine showed a mean decrease in serum prolactin levels whereas patients treated with risperidone showed a mean increase in serum prolactin at week 26.

No interaction between treatment and sex was seen but male patients had a greater mean decrease in prolactin at week 26 compared with the female patients (22.67 mg/dL SE 2.15 vs 8.95 mg/dL SE 2.61, respectively).

#### Correlation between changes from baseline in ASEX Total Score and Serum prolactin levels

There was no meaningful correlation found between the changes from baseline in ASEX Total Score and prolactin (r = 0.12, p = 0.036). Moreover, further inspection of the data revealed some influential outliers. When the two outlying observations were removed from the analyses, it resulted in a loss of statistical significance.

## Discussion

The STAR study was designed to allow comparison of aripiprazole with SOC treatment in a setting close to daily clinical practice, and also to assess the patient and caregiver perspective on the treatment received. The primary outcome measure in the STAR study was the IAQ total score, which takes into account both the efficacy and tolerability of an antipsychotic medication and thus is a measure of effectiveness rather than merely efficacy [21]. The results of STAR demonstrated clearly that at all time points, treatment with aripiprazole was associated with significantly better effectiveness compared with SOC treatment, in the form of one of three atypical agents chosen by the clinician as optimal treatment for the individual patient.

The results of the ASEX evaluation in the STAR study showed that patients treated with aripiprazole experienced significantly less sexual dysfunction at the end of the treatment period compared with patients receiving SOC. These findings are consistent with the existing body of evidence for atypical antipsychotics, as discussed below. When considering the agents within the SOC group individually, olanzapine and quetiapine were associated with less sexual dysfunction at the end of treatment and risperidone was associated with the least improvement in sexual dysfunction, which is in agreement with the Intercontinental Schizophrenia Outpatient Health Outcomes IC-SOHO study [13].

Whilst sexual dysfunction has traditionally run the risk of being underestimated in clinical trials due to the lack of self-reporting and unwillingness to discuss the issue, there is increasing awareness amongst clinicians and allied mental health care professionals that sexual dysfunction is a common and distressing side effect of antipsychotic medication [22]. A survey carried out by the UK patient advocacy group Rethink (formerly the National Schizophrenia Fellowship) showed that 66% of patients who had experienced sexual dysfunction felt that this side effect was 'bad' or 'very bad' [23]. Men appear to suffer more than women [22]. However, in this study female patients showed less improvement in sexual function than their male counterparts who experienced greater improvement in ASEX score. There is evidence that sildenafil is able to address such antipsychotic related sexual dysfunction in men including those patients who also report raised serum prolactin levels. In the study by Gopalakrishnan et al, [4] 77% reported improved erections and 74% (24 patients) felt they would use the medication in future compared with 26% of the placebo group. However, it may be possible to improve sexual dysfunction in both male and female by changing antipsychotic medication rather than adding further medication to the treatment regimen of schizophrenia patients.

Overall, around half of all patients treated with antipsychotics experience sexual dysfunction; the associated distress and frustration may have a profound effect on the quality of life of the patient as well as making personal relationships more difficult, and sexual dysfunction may lead to non-compliance with the antipsychotic treatment regimen [24-26]. In fact, if considering QLS the aripiprazole group reported a greater increase in total QLS and in QLS related to interpersonal relations (table 3) compared to the SOC group. This may be in part related to the greater improvement in ASEX score also observed in these patients.

**Table 3:** Mean change from baseline in QLS Total Score and Subscale Scores at Week 26 (OC)

	**Aripiprazole**		**SE**	**SoC**		**SE**
Total	N = 164	16.21	1.12	N = 162	10.01	1.13
Interpersonal relationships	N = 164	6.34	0.48	N = 162	4.23	0.48
Instrumental role	N = 162	3.14	0.31	N = 161	1.75	0.31
Intrapsychic foundation	N = 164	5.54	0.42	N = 162	3.33	0.42
Common activities	N = 164	1.11	0.12	N = 162	0.73	0.12
Residual symptoms	N = 164	6.65	0.5	N = 162	4.05	0.5

Prolactin plays a key role in the regulation of sexual behaviour and activity [27], and elevated serum prolactin levels associated with antipsychotic medication are known to cause erectile dysfunction, orgasmic difficulties, amenorrhoea and gynaecomastia [11,28]. The serum prolactin measurements also demonstrated that patients in the aripiprazole treated group were more likely to have normal serum prolactin levels at the end of the treatment period compared with patients receiving SOC. Given previous risperidone data, it is not surprising that this was the only agent in the SOC treatment group associated with an increase in mean serum prolactin level at week 26.

The implications of sexual dysfunction for treatment compliance and the prevention of relapse, together with the potential link between primary and secondary prolactinaemia and breast cancer and/or decreased bone mineral density [29] makes serum prolactin an important endpoint in the assessment of antipsychotic therapy. A growing evidence base indicates that, with the exception of risperidone, the atypical antipsychotics are less associated with hyperprolactinaemia than conventional antipsychotics. Some agents, including quetiapine, olanzapine and clozapine, were shown previously to cause no significant or sustained increase in serum prolactin in adult patients [6-10]. More recently, a systematic review of short-term clinical trials with aripiprazole showed that the effect of aripiprazole on serum prolactin levels was comparable to that of placebo, and significantly lower than that of risperidone [14]. The IC-SOHO study demonstrated that a reduction in the incidence of elevated serum prolactin translates to a reduced potential for sexual side effects [13]. Switching patients from conventional antipsychotics or risperidone to an antipsychotic with less potential for prolactin elevation is therefore likely to be an effective measure for improving treatment compliance and reducing the risk of symptomatic relapse in patients experiencing sexual dysfunction during antipsychotic therapy.

One major limitation to the interpretation of the findings is that the STAR study employed an open-label design and both patients and physicians would have been aware as to whether they were receiving study medication or not. Moreover, physicians could choose the SOC medication. This may have introduced bias in subjective measures such as the ASEX, and caution should thus be exercised when interpreting these results.

## Conclusion

In conclusion, the results of this study show that treatment with aripiprazole has the potential to improve not only the clinical effectiveness, but also to reduce the potential for sexual dysfunction, which is increasingly held to be one of the most distressing side effects associated with antipsychotic treatment, as well as the risk of severe side effects associated with hyperprolactinaemia such as loss of bone mineral density and osteoporosis. It is important that a holistic approach is taken to the management of schizophrenia to improve patients' quality of life and potentially compliance.

## Competing interests

Linda Hanssens is an employee of Bristol-Myers Squibb Company. Gilbert L'Italien is an employee of Bristol-Myers Squibb Company. Ronald N. Marcus is an employee of Bristol-Myers Squibb Company. Miranda Pans is an employee of Bristol-Myers Squibb Company. Jean-Yves Loze is an employee of Otsuka Pharmaceutical Development & Commercialization, Inc.

## Authors' contributions

LH and GLI participated in the design of the trial, particularly choosing the research instruments used in the study for patient research and economic outcomes. JYL provided monitoring for the conduct of the study and medical review at the country level. RNM provided validation of the study design and protocol as the clinical research lead. MP participated in the design of the study and performed the statistical analysis. WK performed additional statistical analysis. All authors were involved in preparing the manuscript and, read and approved the final manuscript.

## Pre-publication history

The pre-publication history for this paper can be accessed here:


